# Factors associated with loneliness in patients with diabetes mellitus

**DOI:** 10.1002/nop2.655

**Published:** 2020-10-27

**Authors:** Ewa Kobos, Alicja Szewczyk, Janina Kokoszka‐Paszkot, Beata Dziedzic

**Affiliations:** ^1^ Department of Development of Nursing, Social and Medical Sciences Faculty of Health Sciences Medical University of Warsaw Warsaw Poland; ^2^ Polish Federation for Education in Diabetology Warsaw Poland; ^3^ Clinic of Endocrinology and Diabetology The Children’s Memorial Health Institute Warsaw Poland; ^4^ Specialistic Hospital Gorlice Poland

**Keywords:** adults, diabetes mellitus, loneliness, nursing

## Abstract

**Aim:**

To (a) explore the prevalence of loneliness in patients with diabetes mellitus and (b) identify loneliness‐related factors in the group of hospitalized patients with diabetes mellitus.

**Design:**

A cross‐sectional study.

**Methods:**

The study included 248 patients with diabetes mellitus who were staying in six Polish hospitals. A questionnaire including sociodemographic and clinical data, and the Revised UCLA Loneliness Scale (R‐UCLA), was used for research data collection. Data were collected from March 2019 to June 2019.

**Results:**

Patients with diabetes generally experience moderate loneliness, with almost one‐fifth (16%) of patients experiencing intense loneliness. The patients scored a mean 9.94 out of a possible 20 in belongings and affiliation category and 20.14 out of a possible 40 in the intimate others category. Lower education, being single and the presence of chronic complications of diabetes mellitus were risk factors for increased loneliness.

## 1 INTRODUCTION

Diabetes mellitus is a common chronic disease with a tendency of increasing incidence. Three of four patients with diabetes (352 million people) are at working age (20–64 years). This number is expected to increase up to 417 million by 2030 and 486 million by 2045 (IDF, 2019). Patients with diabetes accounted for 9.1% of adult population in Poland in 2018 (National Health Fund Report, 2019).

Loneliness is a negative state that occurs when a person's social relationships are perceived by that person as quantitatively or qualitatively insufficient (Peplau, 1985). This disturbing emotion occurs in a situation of imbalance between the social needs of an individual and the quality of social relations (Hawkley & Cacioppo, 2010). Data show that about 30 million adults in Europe experience loneliness; poor health, unfavourable economic situation and living alone are associated with increased loneliness indicators (The European Commission's science & knowledge, 2018). Previous studies showed that loneliness is common in the UK (Yang & Victor, 2011). Studies point to an increased risk of mortality in individuals who report high levels of loneliness (Holt‐Lunstad et al., 2015; Rico‐Uribe et al., 2018). Older individuals have a worse perception of their subjective well‐being with increasing levels of loneliness and a growing number of chronic diseases (Tobiasz‐Adamczyk & Zawisza, 2017).

## 2 BACKGROUND

Loneliness has been associated with an increased risk of cardiovascular diseases and stroke (Valtorta et al., 2016), which are the main causes of mortality in patients with diabetes (Emerging Risk Factors Collaboration, 2010). Cardiometabolic factors potentially associated with loneliness may contribute to type 2 diabetes (T2D) (Foti et al., 2020; Hackett & Steptoe, 2017). The mean prevalence of type 2 diabetes mellitus was significantly higher in the lonely group compared with the non‐lonely group in a study assessing the relationship between loneliness and vascular biomarkers in older individuals (O'Luanaigh et al., 2012). Diabetes mellitus and hypertension were important predictors of loneliness in a study assessing the prevalence of loneliness among older patients with non‐infectious diseases (Grover et al., 2019). Research to identify biological mechanisms linking loneliness with poor health shows that the effects of loneliness on health are mediated through the neuroendocrine system (Cacioppo et al., 2015).

An analysis of the relationship between loneliness in the older and metabolic dysregulation showed a fivefold higher potential risk of high HbA1c and threefold higher risk of high BMI among those who experienced loneliness (Shiovitz‐Ezra & Parag, 2019). Scientific evidence suggests a relationship between loneliness and elevated glycated haemoglobin (O'Luanaigh et al., 2012) and metabolic syndrome (Whisman, 2010), both of which are risk factors for T2D.

Diabetes can lead to reduced mobility of the patient, causing difficulties in establishing social contacts, increasing depression and reducing social cohesion among older patients with T2D compared with non‐diabetic patients (Steptoe et al., 2014). It was shown in patients with type 2 diabetes and pre‐diabetes that patients reported an increased sense of isolation and loneliness even in the presence of strong family and social support (McConatha et al., 2020). Lukaschek et al. (2017) showed that low satisfaction with social relationships is associated with an increased risk of diabetes; the prevalence of T2D was higher in socially isolated individuals (Brinkhues et al., 2017).

## 3 METHODS

### 3.1 Aim

To (a) explore the prevalence of loneliness in patients with diabetes mellitus and (b) identify loneliness‐related factors in the group of hospitalized patients with diabetes mellitus.

### 3.2 Research design

A cross‐sectional study.

### 3.3 Participants

The study included 250 hospitalized patients with diabetes mellitus who had no communication problems and who gave their informed consent to participate in the study. The included patients were diagnosed with diabetes at least a year earlier and received pharmacotherapy.

### 3.4 Data collection

Data were collected from March 2019 to June 2019 in diabetes units of six Polish hospitals, during the first or second day of hospitalization. Sociodemographic and clinical data were collected using a questionnaire; the R‐UCLA scale was used for the assessment of loneliness. A total of 248 correctly filled questionnaires were qualified for the analysis.

### 3.5 Instruments

The following tools were used:

#### 3.5.1 A questionnaire

Including a set of sociodemographic data: age, sex, marital status, education level, employment status and the place of residence. Disease‐related variables included the following: the reason for hospital admission, duration and type of diabetes, treatment used and the presence of chronic complications.

#### 3.5.2 Polish version of the Revised UCLA Loneliness Scale (R‐UCLA)

Polish version of the Revised UCLA Loneliness Scale (R‐UCLA) validated by Kwiatkowska et al. (2017) was used in the study. The original version of the scale (UCLA LS) was designed by Russell et al., (1980). The scale consists of 20 statements ranked by patients on a 4‐point scale (1 = I never feel this way, 4 = I often feel this way). The maximum score was 80. The total score consists of the sum of scores from 3 subscales: belongings and affiliation, intimate others and social others. According to Perry's classification system (1990), four degrees of loneliness were defined: a score of 65–80 – a high degree; 50–64 – a moderately high degree; 35–49 – a moderate degree; and 20–34 – a low degree of loneliness.

### 3.6 Data analysis

The normality of the distribution was verified using the Shapiro–Wilk test. Homogeneity of variance was assessed with Levene's test. The following tests were used for hypothesis verification: for a comparison between two groups: *t* test (Student's *t* test) for independent variables or Cochran‐Cox test in the case of failure to meet the assumption of homogeneity of variance; for a comparison between more than two groups: one‐way analysis of variance (along with post hoc RIR Tukey test), Welch's *F* test was used in the case of failure to meet the assumption of homogeneity of variance; non‐parametric Kruskal–Wallis rank ANOVA test (along with the post hoc Dunn's test) was used to compare small samples and highly diverse sample sizes; analysis of correlations: Pearson's linear correlation coefficient. The results were considered statistically significant if the calculated *p*‐value was <.05. Statistica 10.0 was used for statistical analysis.

### 3.7 Ethical issues

The research was performed in accordance with the Declaration of Helsinki. It was voluntary for the patients to answer the questionnaire, and they had the right to withdraw their participation at any time. All the answers were strictly confidential, and the patients were guaranteed full anonymity. Oral informed consent to participate in the study was obtained from participants. The study was approved by the Bioethics Committee of the Medical University of Warsaw.

### 3.8 Trustworthiness

To ensure the validity and reliability of this study, we employed valid and tested tool. In previous analyses, each item had a discriminating power higher than 0.40 and as in the previous and later version, the reliability was perfect (α = 0.94).

## 4 RESULTS

### 4.1 Characteristics of the participants

The mean age of patients was 57.9 years (*SD* 17.4), with the youngest patient aged 18 years, the oldest patient aged 94 years and 52% of patients over the age of 50 years. Marriage/relationship was reported by 71% of respondents; 43% of respondents had secondary education and 15% of patients declared higher education. Retirees and pensioners accounted for 56% of respondents; 35% of respondents were professionally active. Mean duration of diabetes in the study group was 12.14 years (*SD* 9.54), with the longest duration of 42 years. Type 2 diabetes was reported for 70% of patients; 24% of patients received hypoglycaemic agents and insulin therapy. Diabetic neuropathy and retinopathy were reported for 37% and 21% of respondents, respectively; a total of 54% of patients had chronic complications of diabetes. The reasons for admission included uncontrolled diabetes (high glucose levels) in 54.4%, heart diseases in 8.4%, hypertension in 6.4% and diabetic foot syndrome in 4.4% of respondents.

### 4.2 Loneliness

Table 1 presents the findings from the R‐UCLA scale. The mean R‐UCLA score was 38.22 (*SD* =11.55; Med. = 35; Min. = 23; Max. = 76). Low, moderate, high and very high degree of loneliness was reported for 47%, 36%, 13% and 3% of respondents, respectively. The patients obtained a mean score of 9.94 out of a possible 20 (*SD* = 3.05) in belongings and affiliation category and 20.14 out of a possible 40 (*SD =* 7.10) in intimate others category.

**Table 1:** Loneliness in R‐UCLA scale

Category	M	Med.	Min.	Max.	*SD*
Belongings and affiliation	9.94	9.0	5.0	20.0	3.05
Intimate others	20.14	19.0	10.0	40.0	7.10
Social others	8.13	7.0	5.0	20.0	3.82
Total	38.22	35.0	23.0	76.0	11.55

### 4.3 Correlations between loneliness and sociodemographic/clinical factors

As presented in Table 2, higher loneliness was shown in single patients (mean = 41.05; *p* = .025). Significant differences (*p* = .015) were shown between loneliness and the level of education. Patients with primary/junior high school education (mean = 43.72) had higher degree of loneliness compared with those with higher education (mean = 33.71; *p* = .008).

**Table 2:** Comparison of the mean loneliness scores according to the sociodemographic factors

	%	R‐UCLA score		*r*/*t*/*H*	*p*
		Mean	*SD*		
Age (years)	–	–	–	*r* = .09	*p* = .157
Sex					
Male	46	38.94	11.50	*t* = 1.074	*p* = .283
Female	54	37.37	11.60		
Marital status					
Unmarried	14	40.17	13.06	*H* = 6.78	*p* = .079
Married	65	36.62	10.23		
In a relationship	6	42.42	14.39		
Widow/Widower	15	41.86	13.36		
Marital status					
In a relationship	71	37.08	10.68	*t* = −2.26	*p* = .025*
Single	29	41.05	13.15		
Education					
Primary/Junior high school	12	43.72	13.61	*H* = 10.36	*p* = .015*
Vocational	30	38.36	11.21		
Secondary	43	38.27	11.59		
Higher	15	33.71	8.58		
Employment status					
Student	4	34.88	12.11	*H* = 7.45	*p* = .113
Unemployed	5	40.09	16.51		
Employed	35	36.76	11.35		
Retiree	43	39.86	11.20		
Pensioner	13	37.15	11.03		
Place of residence					
City with <50,000 population	31	39.64	12.15	*H* = 5.06	*p* = .167
City with 50,000–200,000 population	12	38.45	11.84		
City with more than 200,000 population	37	36.45	11.46		
Village	20	39.16	10.40		

Note

*t* – *t* test (Student's *t* test); *H* – Kruskal–Wallis test; *r* – Pearson's correlation coefficient; *p* – statistical significance.

* *p* < .05.

Correlations between loneliness and clinical factors are shown in Table 3. Significantly higher loneliness was observed in patients with chronic complications of diabetes (retinopathy, nephropathy and neuropathy) and patients with two or more complications compared with those with one or no complications (*p* = .000). There were no differences in loneliness in the study group in relation to the type of diabetes and the treatment used.

**Table 3:** Comparison of the mean loneliness scores according to the DM characteristics of the patients

	%	R‐UCLA scores		*t*/*F*	*p*
		Mean	*SD*		
Type of DM					
Type 1	30	38.06	13.34	*t* = −0.127	*p* = .898
Type 2	70	38.28	10.73		
Treatment used					
Tablets only	26	36.95	9.76	*F* = 0.507	*p* = .602
Insulin only	50	38.66	12.23		
Tablets + Insulin	24	38.63	11.91		
Retinopathy					
No	79	36.90	10.69	*t* = 3.160	*p* = .002*
Yes	21	43.29	13.36		
Nephropathy					
No	84	37.01	10.56	*t* = −3.127	*p* = .003*
Yes	16	44.47	14.34		
Neuropathy					
No	63	36.19	9.46	*t* = −3.371	*p* = .000*
Yes	37	41.71	13.84		
Diabetic foot syndrome					
No	86	38.27	11.81	*t* = 0.169	*p* = .865
Yes	79	37.91	9.96		
Number of complications					
No complications	46	35.79	9.80	*F* = 8.531	*p* = .000*
1 complication	28	37.18	10.81		
2 or more complications	26	43.70	13.45		
Number of complications					
No complications	46	35.79	9.80	*t* = 3.189	*p* = .001*
Complications	54	40.32	12.54		

Note

*F* – Welch's *F* test; *t* – *t* test (Student's *t* test); *p* – statistical significance.

* *p* < .01.

## 5 DISCUSSION

To the best of our knowledge, this is the first study conducted in Poland to assess loneliness in patients with diabetes mellitus. This study also provides insight in the relationship between sociodemographic and clinical factors and the feeling of loneliness. High levels of loneliness may have a negative impact on diabetic outcomes, while the long‐term and demanding treatment of the disease itself and its potential complications may affect patient's perception of treatment and increase the feeling of loneliness. Studies conducted in a group of patients with chronic diseases showed that the participants perceived their loneliness level as moderate and that their illness perception was negatively affected as their loneliness levels increased (Özkan Tuncay et al., 2018). Loneliness has an impact on self‐management ability in chronically ill individuals (Theeke et al., 2019).

In our study, the average loneliness score obtained by the respondents was 38.22 out of possible 80 (*SD* 11.55). Similar results were obtained in a group of patients with type 2 diabetes (mean = 36.89; *SD* 11.83) in a study which hypothesized that loneliness, as a negative psychosocial stressor, may further affect the already impaired stress response in patients with diabetes (Hackett et al., 2019). These values were slightly lower in a study by Niemcryk et al., (1990) compared with our study, that is mean = 36.67 (*SD* 10.98). However, this study included only patients with type 1 diabetes. Our respondents scored lower compared with patients with cancer (mean = 53.61) (Çıracı et al., 2016; Fanakidou et al., 2018), haemodialysis (Akin et al., 2014), psoriasis (mean = 46.18), chronic leg ulcers (mean = 43.73) (Kouris, Christodoulou, et al., 2016) bullous pemphigoid (mean = 43.01) (Kouris, Platsidaki, et al., 2016) and tuberculosis (mean = 44.36) (Yilmaz & Dedeli, 2016).

The widespread experience of loneliness was confirmed by Hackett et al. (2019). The authors showed that 25% of patients with T2D scored 45 or higher out of a possible 80 on the ULCA scale. This score is higher compared with normative data for the scale: 37.06 for men and 36.06 for women (Russell et al., 1980). In our study, 16% of respondents scored 50 or more, with a mean score of loneliness higher than that reported by Hackett et al., (2019), whose results corresponded with normative values and other behavioural medicine findings (Steptoe et al., 2004).

In our study, low, moderate, moderate high and severe high loneliness was reported for 47%, 37%, 13% and 3% of respondents, respectively, as in accordance with Perry's classification (1990). In a study assessing loneliness in patients with T1D and the relationship between loneliness and perception of treatment, Kusaslan Avci (2018) showed that the percentage of patients with severe high loneliness was higher (4%) and the percentage of patients with moderate high (9.2%) and moderate (21.8%) loneliness was lower compared with our findings. When using total score in the UCLA LS scale for the assessment of the severity of loneliness in older patients with non‐infectious diseases, half of respondents showed moderate (13.2%), moderate high (17.6%) and severe high (18.6%) loneliness. The presence of diabetes was one of the important predictors of loneliness in this study (Grover et al., 2019). In a group of patients with tuberculosis, moderate and high loneliness levels were reported for 49% and 31.2% of respondents, respectively (Yilmaz & Dedeli, 2016).

In this study, patients had a mean score of 9.94 in the belongings and affiliation subscale and 8.13 out of a possible 20 in the social others subscale, and 20.14 out of a possible 40 in the intimate others subscale. Literature review shows that data that would allow for comparison of these findings are missing. As pointed out by adult patients with type 1 diabetes, the disease is present in all aspects of their life and they are dissatisfied with social relationships with regard to diabetes (Joensen et al., 2016). Participants of this study said that they felt “alone” in their everyday life with diabetes, described feeling “excluded from society” and experienced the lack of understanding by friends, family, healthcare workers and society.

It seems that age is an important factor in loneliness. In our study, half of patients were over 50 years of age. According to the data of the National Health Fund, most patients with diabetes in Poland are aged >55 years, with dominant age group 75–84 years in 2018 (National Health Fund Report, 2019). Both, this study and Hackett et al. (2019), showed no significant correlation between loneliness scores and age. The highest cancer‐associated loneliness was observed in young individuals aged 29–39 years; however, it should be noted that the study included patients at the end of life (Çıracı et al., 2016).

Women can be more sensitive to stress in social relationships than men (Cohen‐Mansfield et al., 2016). However, no significant relationship was found between loneliness scores and sex in studies in patients with different chronic diseases (Akin et al., 2014; Russell et al., 1980), which corresponds to our findings and those presented by other authors investigating patients with diabetes (Hackett et al., 2019; Kusaslan Avci, 2018; Niemcryk et al., 1990). In our study, men scored slightly higher than women, which was not confirmed by Kusaslan Avci (2018), who showed that women scored higher than men; however, the difference was not significant. Significantly higher loneliness levels were observed in women with chronic leg ulceration compared with men (Kouris, Christodoulou, et al., 2016).

Previous studies showed a protective effect of marriage on glucose level control, especially in men (Cohen, 2004; Umberson, 1992). An increase in the negative or positive qualities of a marriage may also reduce the prevalence of DM in women and men (Liu et al., 2016; Whisman et al., 2014). Studies on loneliness in patients with chronic diseases also confirmed significantly lower scores in married versus unmarried individuals (Akin et al., 2014; Çıracı et al., 2016; Russell et al., 1980). We believe that a positive marriage reduces loneliness in patients with diabetes, who are likely to be more successful in the long‐term treatment and diabetes self‐control. Hackett et al. (2019) showed that loneliness was significantly correlated with marital status, with lower mean levels of loneliness in married versus single, divorced/separated or widowed individuals. Our study, which also classified patients as single and non‐single, also showed that patients in a relationship experienced lower loneliness. Compared with cancer patients, of whom urban residents experienced significantly higher loneliness (Çıracı et al., 2016), no such a correlation was found in our study.

Several studies investigating loneliness in patients with chronic diseases showed no significant differences between education or employment status and loneliness scores (Akin et al., 2014; Demir, 1989; Russell et al., 1980). However, our study and Kusaslan Avci (2018) showed that significantly lower loneliness scores were obtained by patients with higher education and those professionally active. This probably results from the fact that professionally and socially active patients are less lonely and that higher education level makes it easier to find regular employment. In our study, 35% of patients were professionally active.

Also, there was no significant correlation between loneliness scores and the use of hypoglycaemic agents in both our study and Hackett et al., (2019). Kusaslan Avci (2018) reported higher loneliness levels in patients receiving insulin therapy and those on insulin therapy combined with hypoglycaemic agents. It seems that patients with T1D are particularly susceptible to experiencing negative emotional states associated with diabetes. Previous studies showed that patients with type 1 diabetes experienced significantly higher loneliness (Jones et al., 2016; Kusaslan Avci, 2018; Zhou et al., 2017), which was not confirmed in our study. Patients with type 1 diabetes may experience increased loneliness because of higher stress levels due to treatment regimens and a higher risk of acute complications, such as hypoglycaemia or hyperglycaemic coma. Furthermore, increased loneliness levels in these individuals may affect their readiness and motivation to undergo treatment, which results in worse prognosis. About 40% of women with type 1 diabetes admit experiencing loneliness and social isolation compared with 21% of women with T2D; 34% of women with type 1 diabetes feel less attractive compared with 19% of women with T2D (Barnard & Lloyd, 2012). Although we found no differences in loneliness depending on the type of diabetes in our study, we believe that doctors and other healthcare professionals should monitor loneliness in these patients as part of their everyday clinical practice. Furthermore, preventive measures should be taken, and the importance of this issue should be discussed with patients and their relatives.

Our study showed a relationship between complications and increased loneliness in patients. We did not confirm higher loneliness in patients with diabetic foot syndrome (mean = 37.91) compared with other complications, as shown in another study (mean = 52.25) (Kusaslan Avci, 2018). Diabetic complications may directly affect family, professional and social relationships as a result of physiological and physical stress. Furthermore, reduced compliance with DM treatment in patients with severe loneliness may also increase the risk of diabetic foot syndrome and other diabetic complications. Therefore, we believe that the assessment of loneliness in patients with diabetes may positively contribute to the treatment process. Data show that lonely individuals are more likely to experience somatic and mental health problems, and to develop chronic diseases, high cholesterol levels and diabetes. Loneliness is associated with unhealthy lifestyle (Richard et al., 2017).

## 6 LIMITATIONS

Small sample size and the fact that only hospitalized patients participated in our study are a limitation of our research. The next limitation is that we do not have a comparator non‐diabetic group. Considering the cross‐sectional nature of data, it is impossible to determine the direction of key relationships. Prospective studies are needed to understand whether loneliness is a predictor of poor clinical outcomes and psychosocial functioning indicators in patients with diabetes mellitus.

## 7 CONCLUSION

Our study showed that patients with diabetes generally experience moderate loneliness and one‐fifth of patients experience severe loneliness. Clinical nurses should assess loneliness in patients with diabetes. Patients with chronic complications of diabetes, and those single, less educated and professionally inactive should be monitored more frequently. The knowledge of nurses on psychosocial functioning of patients with diabetes and factors that contribute to loneliness may improve compliance and self‐control of diabetes in patients, and the treatment of diabetes. Nurses can support patients in their sense of coherence to reduce emotional and social loneliness. Healthcare professionals and decision‐makers should develop educational programmes to reduce loneliness as this may potentially delay or minimize complications, and improve health outcomes in patients with diabetes mellitus.

## CONFLICT OF INTEREST

No conflict of interest has been declared by the authors.

## AUTHOR CONTRIBUTIONS

The study idea and study design were conceived by EK and AS. EK, BD and JK‐P wrote the first draft. JK‐P and AS collected the data. EK and BD performed the statistical analyses. All authors have been involved in the interpretation of the results and made important contributions to the drafting of the manuscript. All authors read and approved the final manuscript.

## Data Availability

The date sets used for the current study are available from the corresponding author on reasonable request.
